# Intercostal nerve block is effective in open biopsies of the anterior mediastinal region: Case report and review

**DOI:** 10.1016/j.ijscr.2022.107461

**Published:** 2022-07-26

**Authors:** Marina Brito Gondar, Mariana Fernandes, Pablo Mondragon, Andres Hagerman, Lennart Magnusson

**Affiliations:** a Department of Anesthesiology, Hôpital Fribourgeois, Fribourg, Switzerland; b Faculty of Medicine, University of Fribourg, Fribourg, Switzerland; c Département de Médecine Aiguë, Service d'Anesthésiologie, Hôpitaux Universitaires de Genève, Genève, Switzerland

**Keywords:** Intercostal nerve block, Non-Hodgkin lymphoma, Mediastinal mass, Anterior mediastinum, Loco-regional anesthesia, Case report

## Abstract

**Introduction and importance:**

Anterior mediastinal masses are rare conditions that can become symptomatic through compression of the airways and vascular structures. Fatal or severe complications can occur during anesthesia and surgery. With this review we aim to describe the state of the art in peri-anesthetic management of mediastinal tumors, which we illustrate with a clinical case.

**Presentation of case:**

We report a case of a young female patient suffering from a large anterior mediastinal mass that underwent an open biopsy after intercostal nerve blocks (INB) in six consecutive right intercostal spaces (2nd to 7th). A right anterior mediastinotomy was performed and an excellent analgesic effect was achieved. The patient was awake and did not experience significant pain or cough, having received paracetamol 1 g and returned home later in the day. The diagnosis of non-Hodgkin's lymphoma was later confirmed.

**Discussion:**

Our review showed that anesthesia for mediastinal masses' resection or open biopsy is rare and prone to severe complications. Such complications are more important in children, patients in supine position, under general anesthesia and already symptomatic prior to the procedure. INB presents some advantages against paravertebral block (PVB) and thoracic epidural anesthesia (TEA), is easier to reproduce and has a shorter learning curve. Airway stenting with a rigid bronchoscope can be an alternative.

**Conclusion:**

Multilevel medial axillary line INBs are safer and easier to reproduce than PVB, have less hemodynamic repercussion than TEA and can, therefore, be preferable for open anterior mediastinal biopsies or small masses resection.

## Highlights

• Solo INB for anesthesia in anterior mediastinal masses open procedures is rare.
• Symptomatic patients seem to be more at risk for anesthetic complications.
• INB presents some advantages against PVB or TEA and seems easier to reproduce.

## 1 Introduction

Tumors around the mediastinal cavity are rare [1], [2], [3] and challenging for surgeons and anesthesiologists [4], [5], [6]. These masses are classified as anterior, middle, or posterior mediastinal. Anterior mediastinal tumors cause the most severe and often life-threatening complications related to compression of the airways and vascular structures [1], [5], [7], [8], [9]. Such complications have more potential to degenerate during general anesthesia (GA) [6], [10].

Most mediastinal tumors are symptomatic (chest pain, dyspnea, cough, superior vena cava syndrome (SVCS), etc.) and symptoms are graded mild, moderate, or severe according to patient's tolerance to the supine position [5], [7]. Complication rate during GA is estimated around 7–20% [1], [11] for the anesthetic period and 18 % for the postoperative period [6].

The replacement of GA by locoregional techniques avoids loss of tonus in the airways, trauma to trachea, esophagus or hypopharynx with subsequent oedema, mechanical ventilator-induced lung injury and eventual cardiac or respiratory function compromise that could follow neuromuscular blockers and anesthetic drugs. Thoracic epidural anesthesia (TEA), multilevel intercostal nerve blocks (INB), and paravertebral blocks (PVB) are the most frequent locoregional techniques, used alone or in combination with other techniques through different levels of consciousness [6], [11], [12].

We report a case of a large anterior mediastinal mass that underwent an open biopsy under multilevel INB, while providing a comprehensive literature review and an algorithm proposal for the management of such lesions. We ensure that this report follows the SCARE 2020 criteria [13].

## 2 Presentation of case

**Fig. 1:**
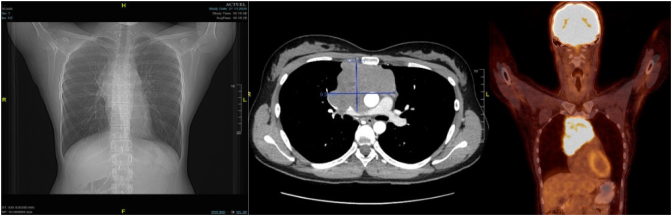
A: Thoracic X-ray antero-posterior view showing a slight enlargement of the upper mediastinum. B: Axial view of a pre-operative CT scan showing the lesion in the anterior mediastinum and an important mass effect. C: Thoracic PET scan in all three orientations showing the hypermetabolic anterior mediastinal lesion.

A 24-year-old female patient, presented with SVCS. An X-Ray showed an enlargement of the mediastinum (Fig. 1). Subsequent computed tomography (CT scan) and positron emission tomography (PET scan) showed an anterior mediastinal mass directly in contact with the pericardium and big vessels (Fig. 1B and C). A mild tracheal deviation and bronchial compression was noted. A transthoracic echocardiographic evaluation revealed a thin pericardial effusion blade with no hemodynamic repercussion. A CT-guided biopsy was inconclusive.

She was scheduled for a right anterior mediastinotomy aiming for a biopsy of the mass. The first and third authors were assigned to the procedure. Midazolam 1 mg I.V. and O_2_ at 6 L/min were administered and the patient was positioned in a slight anti-Trendelenburg (15°). A short hypnosis was performed. An ultrasound (US)-guided INB was repeated in six consecutive right intercostal spaces (2nd to 7th) at the medial axillary line. The US linear probe (Samsung HS60, LA4-18BD) was moved till optimal sagittal views of the ribs, intercostal muscles and parietal pleura were obtained. The needle was inserted in a caudal-to-cranial direction. When the tip of the needle was located under the internal intercostal membrane, at the base of the superior rib, the depression of the parietal pleura by the injection of the local anesthetic confirmed the correct position, after negative aspiration of air or blood. We injected 5 milliliters (ml) of the anesthetic solution (lidocaine 1 % + ropivacaine 0.5 %) per space, with a total of 30 ml. The onset of sensory loss occurred approximately 10 min after the injections. Before starting the incision, 15 cm^3^ of lidocaine 1 % was subcutaneously injected. Electrocautery was used to divide intercostal muscles at the superior board of the inferior rib, the parietal pleura was opened, a retractor was positioned, and multiple biopsy specimens were taken. An excellent analgesic effect was achieved during the operation, and she returned home later that day, comfortable from an analgesic point. The diagnosis of a non-Hodgkin lymphoma was further confirmed.

## 3 Discussion

Current knowledge arises from a couple of randomized control trials (RCT) and retrospective case series of anterior mediastinal masses [14], [15] operated under both GA or locoregional techniques with the latest RCT [15] directly comparing INB, GA and GA with PVB. Previous series, mostly pediatric [2], described between 8 and 20 % rate of severe life-threatening cardiorespiratory complications. Azarow et al. [16] didn't find any difference in mortality between children and adults. Children are probably more susceptible to extrinsic airway obstruction as their airways are more compressible and present a lower vital capacity than adults [16].

Moreover, the aggressivity and duration of the procedures described in the literature is diverse with probably longer resections being performed under GA and open biopsies or debulking under locoregional techniques and thus influencing the estimation of complication rates. To this matter, careful revision of the CT scan angiogram for venous structures compression and review with the surgical team of the operative plan are of utmost importance to anticipate difficulties and complication probability. Other preoperative factor that can help predict severe intraoperative complications is pericardial effusion (even more if an AG or positive-pressure ventilation is added). If it is the case, a pericardial drainage, cardiopulmonary bypass, or both can be performed prior [6].

Hnatiuk et al. [17] postulated that respiratory symptoms showed a trend as indicators of both: the significance of the airway compression and the risk of general anesthesia related complications. Béchard et al. showed a significant correlation between symptoms and perioperative complication rate [5]. These findings suggest that GA shall be avoided whenever possible, particularly in symptomatic patients or in patients with a tracheal area less than 50 % [18]. The same authors also described a sevenfold increase in the risk of post-anesthesia respiratory complications when tracheal compression was greater than 50 % [5] and reported that the risk of complications during and after GA increased 10-fold if the peek expiratory flow rate (PEFR) was reduced by 40 % or less [5]. This contraindicates GA when PEFR is severely reduced or when a mixed pulmonary syndrome is present on pulmonary functional tests (PFT).

Some strategies are proposed in the setting of an extensive and aggressive surgery in patients at risk of having complications under GA: Awake fiber-optic intubation, intubation distal to the airway compression, inhalation induction with spontaneous ventilation, avoidance of muscle relaxants, avoidance of the supine position, positioning changes, and immediate availability of rigid bronchoscopy if needed. For symptomatic patients requiring a diagnostic open biopsy, a locoregional technique shall be used. Another important aspect to consider is eventual phrenic or recurrent laryngeal nerve compression or lesion which may increase the need for post-operative mechanical ventilation.

There are a few locoregional options to obtain proper analgesia. Numerous authors reported on the successful use of TEA to perform several thoracoscopic and open procedures. Also PVB and TEA provide both a good intra- and postoperative pain relief (better than GA according to several retrospective series on other thoracic procedures such as breast surgery) [19]. They allow a better pulmonary function and assessment of the anesthetic impact on this parameter, and fewer pulmonary complications. They can usually be performed at T3 or T4 and show a dense sensitive block that could last until 23 h after administration. But, PVB also has known complications such as epidural spread with paraparesis and Horner triad or pneumothorax (0.5 %). Some mild complications include hypotension (4.6 %), vascular puncture (3.8 %) or pleural puncture (1.1 %) [5], [15]. It is also described some discomfort if a debridement or dissection of some muscular layers is needed. Paravertebral analgesia was associated with approximately 75 % preservation of pre-operative lung function in the first 48 h after surgery compared with 55 % for epidural and intercostal analgesia [11], [15], [17].

When we compare INB with TEA, Mogahed et al. showed that INB can achieve an excellent analgesia while blocking stimulation from the parietal pleura. INB are also better in patients with inadequate cardiovascular reserve [15].

US-guidance in both PVB and INB allows a better accuracy. The paravertebral space (PVS) is a wedge-shaped space lateral to the vertebral column where the spinal nerves emerge from the intervertebral foramina. The lateral paravertebral space tapers and continues laterally into the proximal intercostal space (PICS), a transition that occurs near the costotransverse joint. Both the PVS and PICS contain the intercostal nerves, and the PVS also communicates inferiorly and superiorly with adjacent segmental PVSs, allowing a passage- way for local anesthetic (LA) to spread to several adjacent levels. As the trajectory of the biopsy in this specific case was anterior, a block proximal to the medial axillary line showed to be sufficient without the need to take the risks of coming closer to the spinal canal. The visualization of the intercostal nerves and pleura is also easier in such locations as the thickness of the musculature is less significant. In anterior mediastinal locations like our case, it is our belief that medial INB present fewer risks than PVB for both spinal canal and pleura. It is also an easier technique, for junior consultants or senior staff with less locoregional experience, to reproduce in a safer and confident manner.

## 4 Conclusion

**Fig. 2:**
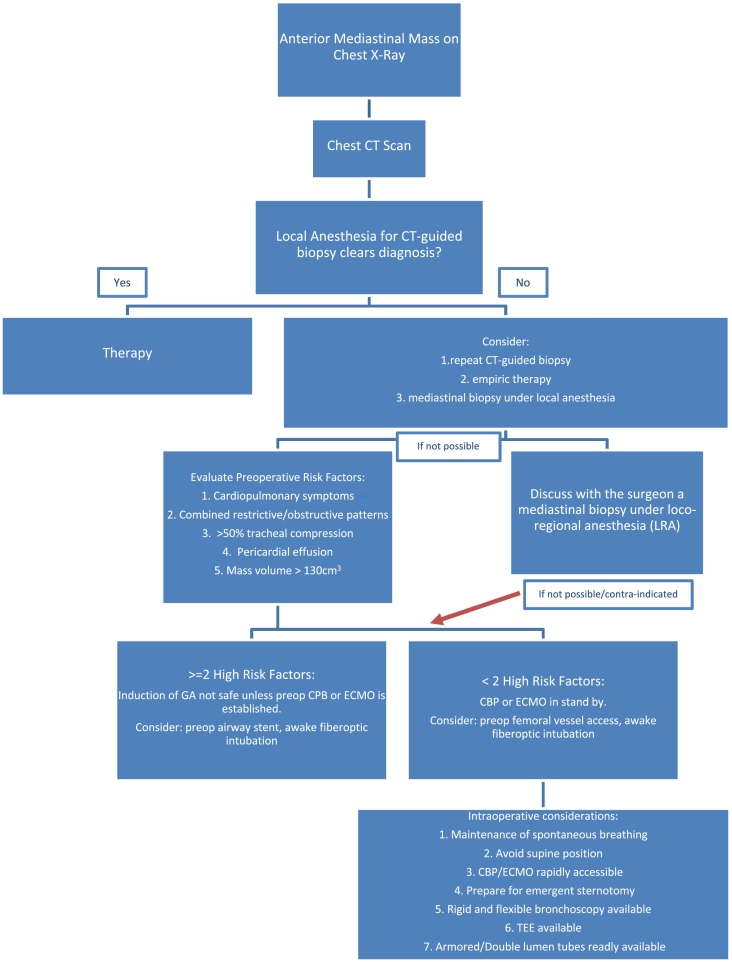
Algorithm for the management of Anterior mediastinal lesions and anesthesia considerations for biopsy procedures. (CPB: cardiopulmonary bypass; ECMO: extracorporeal membrane oxygenation; LRA: loco-regional anesthesia; TEE: transesophageal echocardiogram).

Patients with an anterior mediastinal mass and high risk of perioperative complications can be identified by the presence of cardiorespiratory signs and symptoms at presentation, tracheal compression higher than 50 % or pericardial effusion in the CT scan and combined obstructive and restrictive patterns on PFTs. These findings should prevent the use of GA. Tumor biopsy using a locoregional technique is preferable (Fig. 2).

Maintenance of spontaneous respiration during anesthesia is considered mandatory, particularly for biopsy procedures. Airway stenting, with a rigid bronchoscope in the first instance, is advocated by some. Multilevel INB are safer and easier to reproduce than PVB, have less hemodynamic repercussion than TEA and can, therefore, be preferable for open anterior mediastinal biopsies or small masses' resection.

## Declaration of competing interest

None.
